# Causal Association Between Cholesterol-Lowering Drugs and Diabetic Microvascular Complications: A Drug-Target Mendelian Randomization Study

**DOI:** 10.1155/jdr/3661739

**Published:** 2025-02-28

**Authors:** Bo Yang, Bo Yao, Qu Zou, Sicheng Li, Shun Yang, Mengxue Yang

**Affiliations:** ^1^Department of Endocrinology, Affiliated Hospital of Zunyi Medical University, Zunyi, Guizhou, China; ^2^Department of General Medicine, Affiliated Hospital of Zunyi Medical University, Zunyi, Guizhou, China; ^3^Department of Endocrinology, Shanghai Fifth People's Hospital, Fudan University, Shanghai, China

**Keywords:** cholesterol-lowering, diabetic nephropathy, diabetic neuropathy, diabetic retinopathy, HMGCR, LDL cholesterol, Mendelian randomization, NPC1L1, PCSK9

## Abstract

**Background:** It remains unclear whether cholesterol-lowering therapy can reduce the incidence of microvascular complications in patients with diabetes. We aim to explore the potential causal relationship between three common types of cholesterol-lowering drugs and diabetic microvascular complications through drug-target Mendelian randomization (MR) study, laying the groundwork for the development of new medications.

**Methods:** In this study, we collected single nucleotide polymorphisms (SNPs) associated with HMGCR (3-hydroxy-3-methylglutaryl-CoA reductase) inhibitors, PCSK9 (proprotein convertase subtilisin/kexin type 9) inhibitors, and NPC1L1 (Niemann–Pick C1-Like 1) inhibitors from published genome-wide association study statistics. Subsequently, drug-target MR analyses were performed to investigate the effects of these inhibitors on low-density lipoprotein cholesterol (LDL-C) level–mediated microvascular complications in diabetes mellitus. Coronary atherosclerosis as a positive control. Primary outcomes included diabetic nephropathy, diabetic retinopathy, and diabetic neuropathy from the FinnGen Consortium.

**Results:** The MR analysis revealed significant associations between HMGCR inhibition and increased risks of diabetic nephropathy (OR [95%confidence interval (CI)] = 1.88 [1.50, 2.36], *p* = 5.55 × 10^–8^), retinopathy (OR [95%CI] = 1.86 [1.54, 2.24], *p* = 6.28 × 10^–11^), and neuropathy (OR [95%CI] = 2.63 [1.84, 3.75], *p* = 1.14 × 10^–7^) using the inverse variance weighted method. PCSK9 inhibitors have been associated with an increased risk of diabetic nephropathy (OR [95%CI] = 1.30 [1.07, 1.58], *p* = 0.009) and diabetic neuropathy (OR [95%CI] = 1.40 [1.15, 1.72], *p* = 0.001); NPC1L1 inhibitors significantly reduce the incidence of diabetic retinopathy (OR [95%CI] = 0.48 [0.28, 0.85], *p* = 0.01). The coronary heart disease as positive control.

**Conclusions:** The findings show that HMGCR inhibitors and PCSK9 inhibitors may significantly increase the risk of diabetic microvascular complications. However, NPC1L1 inhibitors may provide protection against diabetic retinopathy.

## 1. Introduction

The International Diabetes Federation (IDF) reports that the number of people with diabetes worldwide is increasing rapidly and is expected to reach 700 million by 2045 [1]. Diabetes mellitus is a chronic metabolic disorder characterized by high blood glucose levels, which can lead to severe microvascular complications [2]. Early epidemiological screening in Taiwan suggested that one-third (36.9%) of diabetes patients may suffer from microvascular complications, and among these microvascular complications, including retinopathy, nephropathy, and neuropathy, diabetic retinopathy is the most common, being found in around 24% of the patients [3]. However, another early epidemiological study conducted in India suggested a prevalence rate of 30.2%, with nephropathy and neuropathy being more common than retinopathy [4]. These complications seriously damage the quality of life of patients with diabetes and are responsible for disability [5–8]. Studies show that dyslipidemia can be seen in more than 60% of diabetes patients [9], and epidemiological studies strongly support dyslipidemia as a risk factor for diabetic microvascular complications [10–13].

Statins, as a first-line drug for lowering cholesterol in patients with diabetes, can effectively reduce the incidence of cardiovascular and cerebrovascular events in patients with diabetes [14]. They can reduce the production of mevalonate by inhibiting HMGCR (3-hydroxy-3-methylglutaryl-CoA reductase) and blocking this enzyme, thus reducing cholesterol synthesis in the liver, especially low-density lipoprotein cholesterol (LDL-C) [15]. However, there are reports that statins may increase blood sugar levels [16, 17]. The mechanism by which statin drugs increase blood glucose levels remains unclear [18]. It remains uncertain whether taking statin drugs after being diagnosed with diabetes reduces the risk of microvascular complications compared to diabetic patients who do not use statin drugs to lower lipid levels [18–21]. Therefore, understanding the causal relationship between statins and diabetic microvascular complications is crucial for optimizing treatment strategies and improving patient outcomes.

Unlike statin medications, PCSK9 (proprotein convertase subtilisin/kexin type 9) inhibitors primarily work by inhibiting the binding of PCSK9 to the low-density lipoprotein receptor (LDLR). This inhibition prevents the degradation of LDLR, allowing more receptors to be available on the surface of liver cells. As a result, more LDL-C is removed from the bloodstream, leading to lower levels of LDL-C [22]. Kosmas et al. [23] conducted the FOURIER (Further cardiovascular OUtcomes Research with PCSK9 Inhibition in subjects with Elevated Risk) trial and found that PCSK9 inhibitors did not increase the risk of diabetes when lowering LDL-C concentrations. However, some studies have shown that PCSK9 inhibitors, when lowering LDL-C levels in patients, also lead to a risk of elevated blood glucose [24, 25].

Niemann–Pick C1-Like 1 (NPC1L1) inhibitors, as a cholesterol absorption inhibitor, can specifically block NPC1L1 and reduce intestinal cholesterol absorption, thereby reducing plasma and liver cholesterol levels [26]. However, there is currently no research indicating a direct association between ezetimibe and diabetic microvascular complications [27]. Notably, a study by Nakamura et al. found that ezetimibe can reduce postprandial hyperlipidemia by reversing insulin resistance [28]. We believe this protective effect may extend to the microvascular health of patients with diabetes.

Due to the contradictory results in existing research, we consider it crucial to conduct additional studies to verify the potential effects of cholesterol-lowering medications on diabetic microvascular complications. Such studies would provide valuable insights into optimizing treatment strategies for managing these complications in diabetic patients. Traditional research methods typically rely on associations observed over time; however, these associations may be confounded by factors such as socioeconomic status, lifestyle, genetics, and others, making it challenging to draw reliable causal inferences [29]. Mendelian randomization (MR) analysis is used as an epidemiological investigation method that allows the analysis of pooled data from genome-wide association studies (GWASs) by means of instrumental variables (IVs) [30]. Due to the randomness arising from genetic variation and the disease-independence of alleles, MR analysis reduces bias caused by confounders and environmental factors, allowing reliable inference of causal relationships between exposures and outcomes [31]. The MR study is based on observational data, although the specific dose and duration information of drug use cannot be obtained directly, but the drug effect can be evaluated indirectly by simulating the effect of the drug on LDL-C by genetic IVs [32]. In this study, we employed MR to explore the causal relationships between cholesterol-lowering drug targets (HMGCR, PCSK9, and NPC1L1) and the risk of developing diabetic microvascular complications (including nephropathy, retinopathy, and neuropathy). By utilizing data from GWAS, we aimed to provide comprehensive insights into how these drug targets influence the progression of diabetic microvascular diseases.

## 2. Methods

### 2.1. Study Design

**Figure 1:**
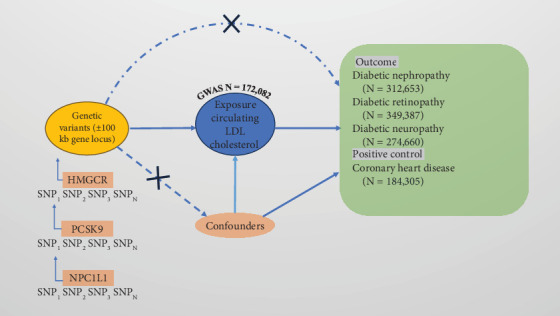
MR study design process for causal relationships between cholesterol-lowering drug targets and diabetic microvascular complications.

This study employs an MR analysis approach to explore the causal relationship between cholesterol-lowering drugs and the risk of diabetic microvascular complications. MR analysis utilizes genetic variants as IVs to estimate the effect of an exposure on outcomes, thereby addressing confounding and reverse causation biases. The data used in this study were obtained from publicly available GWAS databases, and ethical approval was not required. In MR analysis, single nucleotide polymorphisms (SNPs) must meet the following three key assumptions: (1) the relevance assumption, where the IV is strongly associated with the exposure; (2) the independence assumption, where the IV is independent of potential confounders (e.g., obesity, blood glucose, exercise, socioeconomic status, use of ACEI (angiotensin-converting enzyme inhibitor)/ARB (angiotensin receptor blocker), smoking, and hypertension); and (3) the exclusion restriction assumption, where the IV influences the outcome solely through the exposure (Figure 1).

### 2.2. Selection of Genetic Instruments

We selected the SNP associated with the LDL-C level of genome-wide significance (MAF (minor allele frequency) > 1 %) from large-scale GWAS data from the Global Lipids Genetics Consortium (GLGC) as the choice of exposure data. To ensure a robust association between the genetic instruments and the exposure, we set a genome-wide significance threshold of *p* value <5 × 10^–8^. In order to remove the linkage disequilibrium (LD), we set the LD threshold to *r*^2^ = 0.3 and selected the SNPs associated with LDL-C levels within the range of HMGCR, PCSK9, and NPC1L1 loci ±100 kb as the IVs. Finally, we assessed the strength of the association between the selected IVs and the exposure using *F*-statistics. SNPs with an *F*-statistic > 10 were considered valid instruments, while those with weaker associations were excluded to mitigate potential bias arising from weak IVs. Additionally, we identified all SNPs associated with the exposure using the PhenoScanner database (http://www.phenoscanner.medschl.cam.ac.uk/) and removed those associated with confounding factors or outcomes (*p* < 1 × 10^−5^) to avoid potential pleiotropic effects. The remaining SNPs, after excluding confounders, were then used as IVs. The summary data on LDL-C were sourced from the GLGC, comprising 173,082 individuals of European descent. Using these data, drug-target genes associated with LDL-C (HMGCR, PCSK9, and NPC1L1) were identified to simulate the effects of cholesterol-lowering medications. In the end, we obtained 22 SNPs related to HMGCR, 20 SNPs related to PCSK9, and four SNPs related to NPC1L1 (Supporting Information 2: Table S2).

### 2.3. Outcome Sources

We selected three diseases (diabetic nephropathy [more control exclusions], diabetic retinopathy, and diabetic neuropathy) from the FinnGen Consortium Version 9 (https://r9.finngen.fi/) as the outcomes for the drug-target MR analysis in this study. The datasets included 4111 cases of diabetic nephropathy, 308,539 controls, 6818 cases of diabetic retinopathy, 344,569 controls, 2843 cases of diabetic neuropathy, and 271,817 controls. We selected coronary heart disease (CHD) from GWAS summary statistics as a positive control, which included data from 60,801 cases and 123,504 controls. These datasets are all from European populations (Supporting Information 1: Table S1).

### 2.4. Data Analysis

The statistical analysis in this study utilized the MR analysis framework to determine the causal associations between cholesterol-lowering drugs and diabetic microvascular complications. Initially, the genetic variants associated with the drug targets HMGCR, PCSK9, and NPC1L1 were extracted from published GWAS. The SNPs were harmonized with outcome datasets to ensure the alignment of alleles. Various MR methods, including MR-Egger, weighted median, inverse variance weighting (IVW), simple mode, weighted mode, and MR-PRESSO (MR pleiotropy residual sum and outlier test), were applied to verify the consistency of the results. The IVW method is considered to be one of the most important methods in MR analysis, which estimates the causal effect of exposure on results by weighted genetic tools so as to evaluate the effect of exposure on results more accurately [33–35]. For heterogeneity assessment, Cochran's *Q* test was used, with a *p* value > 0.05 indicating no significant heterogeneity. Horizontal pleiotropy, which could confound the causal inference, was evaluated using the MR-Egger regression intercept. A *p* value > 0.05 was considered indicative of no horizontal pleiotropy [36]. SNPs that were directly associated with the outcome were excluded. Sensitivity analyses included the leave-one-out method, which tested the impact of each SNP individually on the overall results to ensure robustness. Additionally, the MR-PRESSO test was employed to detect and correct for outliers, further refining the causal estimates [37] (Supporting Information 3: Table S3). To validate the IVs, we used summary data from a GWAS of CHD as a positive control. The statistical analyses were conducted using R software (Version 4.3.3), specifically employing the TwoSampleMR and MR-PRESSO packages.

## 3. Results

### 3.1. Positive Control Analysis

**Figure 2:**
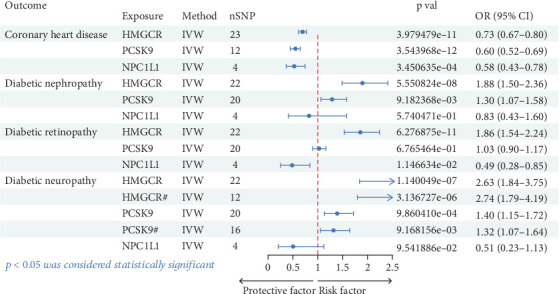
The forest plot showed the causal associations between exposures (drug target) and outcomes (diabetic microvascular complications) in the MR analysis. nSNP, number of single nucleotide polymorphisms; OR, odds ratio; CI, confidence interval.

The positive control analysis assessed the association between cholesterol-lowering drug targets and coronary atherosclerosis to validate our MR approach. The analysis conducted through the IVW method indicates that statins (OR [95%confidence interval (CI)] = 0.73 [0.67, 0.80)], *p* = 3.98 × 10^–11^), PCSK9 inhibitors (OR [95%CI] = 0.65 [0.57, 0.73], *p* = 6.44 × 10^–12^), and NPC1L1 inhibitors (OR [95%CI] = 0.58 [0.43, 0.78], *p* = 0.0003) significantly reduce the risk of CHD (Table 1 and Figure 2). These results confirmed the expected benefits of cholesterol-lowering therapies on cardiovascular outcomes, reinforcing the validity of our methods and providing confidence in the causal inferences drawn for diabetic microvascular complications.

### 3.2. Primary Analysis

This study evaluated the associations between genetic proxies of cholesterol-lowering drug targets (HMGCR, PCSK9, and NPC1L1) and the risk of diabetic complications, including nephropathy, retinopathy, and neuropathy. Using IVW analysis, the results demonstrated significant differences in the effects of these targets across outcomes. For diabetic nephropathy, HMGCR inhibition was associated with an increased risk (OR [95%CI] = 1.88 [1.50, 2.36], *p* = 5.55 × 10^–8^). Similarly, PCSK9 inhibition showed a moderate increase in risk (OR [95%CI] = 1.30 [1.07, 1.58], *p* = 0.009). NPC1L1 inhibition did not exhibit a statistically significant effect (*p* = 0.574). For diabetic retinopathy, HMGCR inhibition showed a strong association with higher risk (OR [95%CI] = 1.86 [1.54, 2.24], *p* = 6.28 × 10^–11^). PCSK9 inhibition had no significant effect (*p* = 0.677), while NPC1L1 inhibition was associated with a reduced risk (OR [95%CI] = 0.48 [0.28, 0.85], *p* = 0.011). For diabetic neuropathy, HMGCR inhibition had the strongest association, showing a substantial increase in risk (OR [95%CI] = 2.63 [1.84, 3.75], *p* = 1.14 × 10^–7^). PCSK9 inhibition was moderately associated with increased risk (OR [95%CI] = 1.40 [1.15, 1.72], *p* = 0.001). NPC1L1 inhibition demonstrated no statistically significant effect (*p* = 0.095). These findings highlight the differential effects of cholesterol-lowering targets on diabetic complications, suggesting potential considerations for therapeutic strategies (Table 1 and Figure 2).

### 3.3. Sensitivity Analysis

Cochran's *Q* test and MR-Egger regression were used to confirm the robustness and reliability of our primary findings (Supporting Information 3: Table S3 for details) [38, 39], where *p* > 0.05 for Cochran's *Q* test indicates no significant heterogeneity and *p* > 0.05 for the MR-Egger regression equation indicates no horizontal pleiotropy. In this study, we found significant heterogeneity between HMGCR and diabetic neuropathy (*p*_Cochran's *Q* test_ = 0.032; Table 1 for details). In addition, there was horizontal pleiotropy between PCSK9 and diabetic neuropathy (*p*_Egger's⁣intercept_ = 0.040; Table 1 for details). To mitigate the impact of heterogeneity on the study results, I adjusted the LD parameter from *r*^2^ = 0.3 to *r*^2^ = 0.1 by selecting more stringent SNPs to reduce the heterogeneity of the IVs and then performing MR analysis again. Excitingly, after adjusting the LD parameter, significant heterogeneity was eliminated (*p* > 0.05; Supporting Information 3: Table S3 for details). Ultimately, none of our sensitivity analyses showed heterogeneity or horizontal pleiotropy (all *p* > 0.05; Supporting Information 3: Table S3). Additionally, through leave-one-out analysis, it was found that removing one SNP at a time did not alter the overall effect, which remained consistent with the primary effect observed with all SNPs included. These sensitivity analyses confirm the robustness and reliability of our primary findings.

## 4. Discussion

This study employed data on diabetic microvascular complications from the Finnish database. Through MR analysis of these complications, we examined the effects of three common cholesterol-lowering drug targets (HMGCR, PCSK9, and NPC1L1) on three major diabetic microvascular complications: diabetic retinopathy, diabetic nephropathy, and diabetic neuropathy. Our findings indicate that HMGCR inhibitors were significantly associated with an increased risk of diabetic nephropathy, diabetic retinopathy, and diabetic neuropathy. PCSK9 inhibitors increase the risk of diabetic nephropathy and diabetic neuropathy. Furthermore, NPC1L1 inhibitors can reduce the risk of diabetic retinopathy. These results suggest that statins and PCSK9 inhibitors may increase the risk of diabetic microvascular complications, while NPC1L1 inhibitors may provide protection against diabetic retinopathy.

Statins have long been supported by extensive evidence for reducing the risk of atherosclerotic cardiovascular disease (ASCVD) in patients with diabetes [40, 41]. Notably, some studies have suggested that statin therapy may increase blood glucose levels, thereby elevating the risk of new-onset diabetes (NOD), particularly in patients with risk factors such as metabolic syndrome or obesity [17–19, 42]. However, the role of statins in the progression of microvascular complications in diabetic patients remains unclear. Research by Nielsen and Nordestgaard indicated that prediagnosis statin use was not associated with an increased risk of microvascular complications [43]. In contrast, a subsequent MR analysis suggested that statins might be a risk factor for diabetic nephropathy and retinopathy [44], a finding consistent with our study. However, some studies have shown that statins with different dosages and potencies have varying effects on the kidneys [45–47]. de Zeeuw et al. [45] found that 80 mg/days of rosuvastatin increased the risk of proteinuria, but serum creatinine decreased when the dose was adjusted to 40 mg/day. Studies have shown that rosuvastatin, as a highly hepatoselective drug associated with poor renal tubular cell uptake, does not generally cause proteinuria [48], and if this is the case, then moderately potent, less hepatoselective, and potent inhibitors of albumin uptake like simvastatin and fluvastatin may be more likely to cause proteinuria than rosuvastatin [46]. A study of Korean patients with dyslipidemia and Type 2 diabetes mellitus (T2DM) in South Korea found that pravastatin significantly improved eGFR (estimated glomerular filtration rate) in patients with T2DM and dyslipidemia and had no significant fluctuations in blood sugar in patients with diabetes [49]. A recent study suggested that statin initiation in patients with T2DM is associated with a lower risk of kidney disease development [50]. However, a National Health and Nutrition Survey (NHANES) study of Americans shows that long-term use of statins increases the risk of diabetic nephropathy, especially simvastatin and atorvastatin [51]. In addition, a PREVEND (prevention of end-stage renal and vascular disease) intervention trial in subjects with microalbuminuria showed that pravastatin (40 mg) did not significantly reduce urinary albumin excretion [52].

Currently, fibrate medications have gained broader recognition for their benefits in reducing the risk of diabetic retinopathy [53], while the role of statins remains controversial [54]. Our study found that statin use may exacerbate the risk and progression of diabetic retinopathy. However, due to the limited number of IVs and the lack of control, a more thorough explanation of the underlying mechanisms could not be provided. In contrast, a recent MR analysis using the NHANES database, along with GWAS and eQTL (expression quantitative trait locus) data, more comprehensively demonstrated a causal relationship between statin use and the significant increase in the risk of diabetic retinopathy [55], which is consistent with our findings. A meta-analysis and systematic review indicated that cholesterol-lowering agents, including statins, show a protective effect on diabetic retinopathy progression and might be associated with reduced risk in the development of diabetic macular edema (DME) [56]. Additionally, a cohort study has also indicated that statin use in patients with T2DM can reduce the risk of DME and the need for diabetic retinopathy treatments, such as laser therapy [57]. However, in patients with Type 1 diabetes mellitus (T1DM), the effect of statins on diabetic retinopathy has not been confirmed. Diabetic neuropathy is a complex, multifactorial microvascular complication in which diabetic peripheral neuropathy (DPN) is the most common. Some studies have shown that statins may aggravate neuropathy [58, 59], while others have improved [60, 61]. However, a clinical trial conducted by Davis et al. [62] found that the incidence of DPN in T2DM patients treated with statins decreased by 35%. Furthermore, a double-blind, placebo-controlled trial also showed that simvastatin/ezetimibe and rosuvastatin significantly reduced lipid peroxidation in patients with DPN [63]. Our results show that statins increase the risk of diabetic microvascular complications, which contradicts some previous studies, indicating that the mechanism by which statins affect blood glucose is not clear. Therefore, further experiments are needed to clarify the complex relationship between them.

PCSK9 inhibitors are popular cholesterol-lowering drugs in recent years. Compared with statins alone, these inhibitors can reduce LDL-C levels by 50% and 70% [64]. Previous studies have emphasized the cardiovascular benefits of PCSK9 inhibitors but have not extensively addressed their effects on microvessels [65, 66]. However, it has been reported that elevated PCSK9 levels may aggravate the vascular consequences and prognosis associated with diabetes [67]. An animal experiment conducted by Feng et al. [68] found that PCSK9 triggers mitochondrial DNA damage and activates the cyclic GMP-AMP synthase (cGAS)-stimulator of interferon genes (STING) pathway in diabetic nephropathy, which can effectively reduce inflammation and delay its progression. Recent studies on the mtDNA-cGAS-STING signaling pathway and the treatment of diabetic nephropathy have also found its key role in this condition [69, 70]. In addition, through the study of the cGAS-STING signal pathway, some scholars have also found its role in retinal microvascular endothelial cells (RMECs), which provides a new method to reduce the inflammatory response of diabetic retinal diseases [71]. In a recent animal study, we found that alirocumab improved nerve conduction, morphological changes, and small fiber deficits in DPN rats [72]. It is reported that *α*-lipoic acid (ALA) is considered to be a promising first-line disease relief and antioxidant therapy for DPN, which can prevent the early development and progress of DPN by exerting direct and indirect antioxidant effects [73]. Supplementation of ALA can effectively reduce serum PCSK9 concentration by 70%, and these effects are related to a significant decrease in the number of low-density lipoprotein (LDL) particles and an increase in the number of LDLRs in the liver [74]. Therefore, the regulatory effect of ALA on PCSK9 may increase the clearance rate of LDL in blood circulation, thereby reducing cholesterol levels. However, our study contradicts previous evidence that PCSK9 inhibitors do not increase the risk of diabetes [75, 76]. These conflicting findings challenge the interpretation of PCSK9 inhibitors in the treatment of diabetic microvascular complications and emphasize the importance of further research to verify this claim.

It is worth noting that our study found the protective effect of NPC1L1 inhibitors on diabetic retinopathy. However, a meta-analysis conducted by Lotta et al. [77] showed that exposure to LDL-C-lowering genetic variants NPC1L1 was associated with an increased risk of T2DM. In contrast, an animal study showed that NPC1L1 inhibitors reduced diet-induced hyperglycemia and insulin resistance [78]. In addition, some research reports have pointed out that the reason why ezetimibe does not show an effect on diabetes, in addition to its own cholesterol-lowering mechanism, may also be related to the other “on-target” and “off-target” effects of these drugs [79]. Our investigation only revealed the causal relationship between NPC1L1 and diabetic retinopathy but had no statistical significance for diabetic nephropathy and diabetic neuropathy. These results suggest a complex relationship between the mechanism leading to the reduction of LDL-C and diabetic microvascular complications.

This study used MR analysis to explore the causal relationship between cholesterol-lowering drug targets and diabetic microvascular complications, effectively addressing biases such as confounding and reverse causation [80]. By leveraging genetic variants as IVs, we ensured more reliable causal inferences. The large GWAS datasets, comprising over 172,000 individuals for LDL-C and over 300,000 for diabetic complications, provided substantial statistical power and validity. We applied multiple MR methods, including IVW, MR-Egger, weighted median, simple mode, and weighted mode, to confirm the robustness of our findings [81–83]. The results showed significant causal relationships between HMGCR inhibition and increased risks of diabetic nephropathy, retinopathy, and neuropathy, consistently across various MR methods. In contrast, PCSK9 inhibition was associated with a moderate increase in risk for nephropathy and neuropathy, but not retinopathy. This detailed methodological approach and comprehensive dataset provide a nuanced understanding of how different cholesterol-lowering targets affect diabetic microvascular disease, offering valuable insights for clinical practice and future research.

Despite the strengths of this study, several limitations should be acknowledged. Firstly, due to the lack of individual-level clinical diagnostic data in the publicly available GWAS database, we were unable to assess the diagnostic accuracy or clinical severity of microvascular complications; thus, further validation of the clinical relevance of our findings is needed. Secondly, while MR reduces the risk of confounding, it is not entirely free from bias, particularly if the genetic variants used as IVs exhibit pleiotropy, affecting multiple traits. Thirdly, the causal estimates rely on the assumption that the selected SNPs influence the outcomes solely through their effect on LDL-C, which might not fully capture the complexity of lipid metabolism and its interactions with other biological pathways. Due to the limitations of available GWAS data, our study focused on European populations, which may limit the generalizability of our findings to other regions. Ethnic differences, particularly in East Asian populations, may affect the relationship between triglycerides and diabetes complications, influenced by genetic, lifestyle, and dietary factors. Since Asian populations were not included, further research is needed to explore ethnic variations and their impact on cholesterol-lowering treatments, providing evidence for personalized treatment strategies. Additionally, the limited number of IVs (HMGCR, PCSK9, and NPC1L1) may introduce some degree of bias or uncertainty to our results. Future studies that incorporate a broader range of IVs are expected to provide more reliable and comprehensive insights. Investigate the broader metabolic effects of cholesterol-lowering therapies beyond LDL-C, including high-density lipoprotein (HDL) and triglycerides, to provide a more holistic understanding of their impact on diabetic complications. By addressing these limitations and expanding the scope of analysis, future research can further elucidate the intricate relationships between cholesterol-lowering therapies and diabetic microvascular complications, ultimately improving therapeutic strategies and patient outcomes.

## 5. Conclusions

After drug-targeting MR analysis, our results suggest that HMGCR inhibitors and PCSK9 inhibitors may significantly increase the risk of diabetic microvascular complications. However, NPC1L1 inhibitors may provide protection against diabetic retinopathy.

## Figures and Tables

**Table 1:** The association between LDL cholesterol, mediated by the genes and diabetic microvascular complications, was examined using the IVW method.

**Outcome**	**Target**	**Method**	**nSNP**	**p** ** val**	**OR (95% CI)**	**p**_**Cochran's**_⁣_**Q**__**test**_	**p**_**Egger's intercept**_	**p**_**MR-PRESSO global**_
Coronary heart disease	HMGCR	IVW	23	3.98e − 11	0.73 (0.67–0.80)	0.584	0.051	0.595
	PCSK9	IVW	12	3.54e − 12	0.60 (0.52–0.69)	0.109	0.757	0.130
	NPC1L1	IVW	4	0.000345063	0.58 (0.43–0.78)	0.918	0.552	0.921

Diabetic nephropathy	HMGCR	IVW	22	5.55e − 08	1.88 (1.50–2.36)	0.582	0.385	0.647
	PCSK9	IVW	20	0.009182368	1.30 (1.07–1.58)	0.112	0.053	0.137
	NPC1L1	IVW	4	0.574047114	0.83 (0.43–1.60)	0.404	0.303	0.404

Diabetic retinopathy	HMGCR	IVW	22	6.28e − 11	1.86 (1.54–2.24)	0.352	0.464	0.416
	PCSK9	IVW	20	0.676546367	1.03 (0.90–1.17)	0.981	0.787	0.988
	NPC1L1	IVW	4	0.011466344	0.49 (0.28–0.85)	0.316	0.301	0.386

Diabetic neuropathy	HMGCR	IVW	22	1.14*e* − 07	2.63 (1.84–3.75)	0.032	0.392	0.048
	HMGCR⁣^∗^	IVW	12	3.14*e* − 06	2.74 (1.79–4.19)	0.213	0.647	0.308
	PCSK9	IVW	20	0.000986041	1.40 (1.15–1.72)	0.905	0.040	0.769
	PCSK9⁣^∗^	IVW	16	0.009168156	1.32 (1.07–1.64)	0.866	0.230	0.761
	NPC1L1	IVW	4	0.095418857	0.51 (0.23–1.13)	0.664	0.525	0.641

*Note:* Asterisk (∗) represents SNPs selected when the linkage disequilibrium (LD) parameter changes from *r*^2^ < 0.3 to *r*^2^ < 0.1.

Abbreviations: HMGCR, 3-hydroxy-3-methylglutaryl-CoA reductase; NPC1L1, Niemann–Pick C1-Like 1; PCSK9, proprotein convertase subtilisin/kexin type 9.

## Data Availability

The data and materials used in this study are available upon reasonable request from the corresponding author. Access to the FinnGen dataset can be obtained through their official data access policies and procedures. Requests for specific datasets or materials utilized in this research should be directed to the corresponding author for consideration and further instructions.

## Nomenclature

HMGCR: 3-hydroxy-3-methylglutaryl-CoA reductase
PCSK9: proprotein convertase subtilisin/kexin type 9
MR: Mendelian randomization
SNP: single nucleotide polymorphism
GWAS: genome-wide association study
LDL-C: low-density lipoprotein cholesterol
HDL: high-density lipoprotein
IVW: inverse variance weighted
LD: linkage disequilibrium
OR: odds ratio
CI: confidence interval
NPC1L1: Niemann–Pick C1-like 1

## Supporting Information

Supporting Information 1 Table S1: information of GWAS summary data.

Supporting Information 2 Table S2: the detail of the instrumental variable corresponding.

Supporting Information 3 Table S3: the effect of HMGCR, PCSK9, and NPC1L1 inhibitors on microvascular complications of diabetes.
