# Supplementary material for: Causal Association Between Cholesterol-Lowering Drugs and Diabetic Microvascular Complications: A Drug-Target Mendelian Randomization Study
Source: J Diabetes Res. 2025 Feb 28;2025:3661739. doi: 10.1155/jdr/3661739 (PMC11986941; doi:10.1155/jdr/3661739)
Supplement: Supporting Information 1 — Table S1: information of GWAS summary data. [file 3661739.f1.pdf]

Table S1. Information of GWAS summary data.

Characteristic	Resource	Sample size	Population ancestry	Data download
**GWAS summary data**				
Diabetic nephropathy	FinnGen Consortium	Number of cases:4,111<br>Number of controls: 308,539	European	https://storage.googleapis.com/finngen-public-data-r9/summary_stats/finngen_R9_DM_NEPHROPATHY_EXMORE.gz
Diabetic retinopathy	FinnGen Consortium	Number of cases:6,818<br>Number of controls: 344,569	European	https://storage.googleapis.com/finngen-public-data-r9/summary_stats/finngen_R9_H7_RETINOPATHYDIAB.gz
Diabetic neuropathy	FinnGen Consortium	Number of cases:2,843<br>Number of controls: 271,817	European	https://storage.googleapis.com/finngen-public-data-r9/summary_stats/finngen_R9_DM_NEUROPATHY.gz
LDL cholesterol	Global Lipids Genetics Consortium	Number of cases and controls:173,082	Predominantly European	http://csg.sph.umich.edu/willer/public/lipids2013/
Coronary heart disease	UK Biobank	Number of cases: 60,801<br>Number of controls: 123,504	Mixed	https://gwas.mrcieu.ac.uk/datasets/ieu-a-7/
