# Supplementary material for: Causal Association Between Cholesterol-Lowering Drugs and Diabetic Microvascular Complications: A Drug-Target Mendelian Randomization Study
Source: J Diabetes Res. 2025 Feb 28;2025:3661739. doi: 10.1155/jdr/3661739 (PMC11986941; doi:10.1155/jdr/3661739)
Supplement: Supporting Information 2 — Table S2: the detail of the instrumental variable corresponding. [file 3661739.f2.pdf]

Table S2. The detail of instrumental variable corresponding.

SNP	Chr	Pos	Effect allele	Other allele	Effect allele frequency	Beta	SE	Pval	F-statistic
**HMGCR**									
rs10055011	5	74798156	A	G	0.1319	0.0642	0.0056	1.02E-26	131.4290062
rs10056022	5	74969415	A	G	0.08575	0.0424	0.0066	1.87E-10	41.27062672
rs10066707	5	74560579	A	G	0.4169	0.0497	0.0054	2.97E-19	84.70761999
rs10069744	5	74263455	C	T	0.1069	0.043	0.0059	6.83E-12	53.11658064
rs10515198	5	74641560	A	G	0.1029	0.0599	0.0061	5.99E-22	96.42534393
rs12659791	5	74757758	C	T	0.1557	0.0433	0.005	1.42E-18	74.99512026
rs12916	5	74656539	C	T	0.4314	0.0733	0.0038	7.79E-78	372.0814148
rs16872670	5	74929312	A	G	0.05673	0.0553	0.0084	1.80E-09	43.34000053
rs16872768	5	74982207	C	T	0.3852	0.0463	0.0038	4.87E-33	148.4540365
rs2035191	5	74413713	C	T	0.186	0.0611	0.0047	5.70E-35	168.9989189
rs2216572	5	74972877	A	G	0.1042	0.0371	0.0064	2.59E-08	33.6035448
rs3804231	5	74696779	A	G	0.1319	0.0642	0.0053	1.88E-29	146.7288585
rs3857388	5	74620377	C	T	0.128	0.0421	0.0059	2.20E-11	50.9163649
rs3935470	5	74352180	G	A	0.3839	0.0439	0.0039	1.59E-27	126.7059613
rs4382144	5	74274675	A	G	0.5871	0.0289	0.0037	3.25E-15	61.00837525
rs5744578	5	74840745	G	A	0.1042	0.0427	0.0069	5.25E-09	38.29612133
rs5744672	5	74877803	C	T	0.4169	0.0654	0.0037	4.53E-64	312.427512
rs5744707	5	74890618	G	A	0.1029	0.0549	0.0061	5.85E-19	80.99948185
rs72633962	5	74569028	C	T	0.1412	0.06	0.0072	3.33E-15	69.44400021
rs7717355	5	74951901	A	G	0.1293	0.0453	0.0061	2.32E-13	55.14853192
rs7727150	5	75132546	G	T	0.4789	0.024	0.0038	4.97E-09	39.88894151
rs904743	5	74917862	G	A	0.1372	0.0673	0.0054	4.39E-31	155.3244522
**HMGCR**[#]									
rs10069744	5	74263455	C	T	0.1069	0.043	0.0059	6.83E-12	53.11653364
rs10474429	5	74417980	A	G	0.2533	0.0324	0.0064	3.06E-08	25.62871963
rs12916	5	74656539	C	T	0.4314	0.0733	0.0038	7.79E-78	372.0810856
rs16872670	5	74929312	A	G	0.05673	0.0553	0.0084	1.80E-09	43.33996219
rs16872768	5	74982207	C	T	0.3852	0.0463	0.0038	4.87E-33	148.4539051
rs17563312	5	74949600	T	A	0.05937	0.0486	0.0077	1.45E-09	39.83711926
rs2035191	5	74413713	C	T	0.186	0.0611	0.0047	5.70E-35	168.9987694
rs3857388	5	74620377	C	T	0.128	0.0421	0.0059	2.20E-11	50.91631984
rs4382144	5	74274675	A	G	0.5871	0.0289	0.0037	3.25E-15	61.00832127
rs5744672	5	74877803	C	T	0.4169	0.0654	0.0037	4.53E-64	312.4272356
rs7727150	5	75132546	G	T	0.4789	0.024	0.0038	4.97E-09	39.88890621
rs9942407	5	74540187	A	G	0.4182	0.0489	0.0053	3.13E-19	85.12611562
**PCSK9**									
rs10493176	1	55538552	G	T	0.1148	-0.0776	0.0102	2.54E-14	57.87885574
rs11206510	1	55496039	C	T	0.1544	-0.0831	0.005	2.38E-53	276.2223886
rs11206514	1	55516004	A	C	0.6108	0.0507	0.0041	9.95E-33	152.9132232
rs11583974	1	55551718	A	G	0.03034	0.0646	0.0117	3.95E-09	30.48527732
rs11591147	1	55505647	T	G	0.01715	-0.497	0.018	8.57E-143	762.3679054
rs12066643	1	56240278	T	C	0.1187	-0.0389	0.0064	1.06E-08	36.9433345
rs12067569	1	55528629	A	G	0.0343	0.0885	0.01	1.97E-17	78.32192968
rs1475701	1	55638546	C	T	0.03562	0.0904	0.0092	1.46E-20	96.55128181
rs1874776	1	55743519	C	T	0.7823	0.044	0.0044	2.76E-21	99.99927183
rs207145	1	55808143	T	C	0.8905	0.0495	0.0057	6.19E-18	75.41496331
rs2479394	1	55486064	A	G	0.715	-0.0386	0.0041	1.58E-19	88.63469069
rs2479409	1	55504650	A	G	0.6675	-0.0642	0.0041	2.51E-50	245.1879826
rs2495495	1	55496556	C	T	0.8654	-0.0342	0.0059	3.52E-08	33.60044479
rs2647281	1	55724704	G	A	0.05541	0.0589	0.0095	2.27E-09	38.43972009
rs4927193	1	55509872	C	T	0.1306	-0.0352	0.0056	4.27E-11	39.50991638
rs4927207	1	55713628	A	G	0.1715	-0.0692	0.0049	2.36E-39	199.4421129
rs572512	1	55517344	T	C	0.3456	0.0478	0.0047	5.31E-26	103.4324745
rs585131	1	55524116	T	C	0.8153	0.0637	0.005	2.70E-35	162.3064181
rs6662286	1	55730327	C	T	0.90633	0.0989	0.0073	6.30E-36	183.5454828
rs7536204	1	55666477	A	C	0.5884	0.0418	0.0054	5.39E-15	59.9186309
**PCSK9**[#]									
rs10493176	1	55538552	G	T	0.1148	-0.0776	0.0102	2.54E-14	57.87885574
rs10888896	1	55509213	C	G	0.7203	0.0426	0.0049	2.14E-14	75.58295649
rs11206510	1	55496039	C	T	0.1544	-0.0831	0.005	2.38E-53	276.2223886
rs11583974	1	55551718	A	G	0.03034	0.0646	0.0117	3.95E-09	30.48527732
rs11591147	1	55505647	T	G	0.01715	-0.497	0.018	8.57E-143	762.3679054
rs12066643	1	56240278	T	C	0.1187	-0.0389	0.0064	1.06E-08	36.9433345
rs12067569	1	55528629	A	G	0.0343	0.0885	0.01	1.97E-17	78.32192968
rs1475701	1	55638546	C	T	0.03562	0.0904	0.0092	1.46E-20	96.55128181
rs207145	1	55808143	T	C	0.8905	0.0495	0.0057	6.19E-18	75.41496331
rs2479394	1	55486064	A	G	0.715	-0.0386	0.0041	1.58E-19	88.63469069
rs2479409	1	55504650	A	G	0.6675	-0.0642	0.0041	2.51E-50	245.1879826
rs2647281	1	55724704	G	A	0.05541	0.0589	0.0095	2.27E-09	38.43972009
rs4927207	1	55713628	A	G	0.1715	-0.0692	0.0049	2.36E-39	199.4421129
rs585131	1	55524116	T	C	0.8153	0.0637	0.005	2.70E-35	162.3064181
rs6662286	1	55730327	C	T	0.90633	0.0989	0.0073	6.30E-36	183.5454828
rs7552841	1	55518752	T	C	0.3654	0.0368	0.0044	5.40E-15	69.94990386
**NPC1L1**									
rs2073547	7	44582331	G	A	0.1939	0.0485	0.0049	1.92E-21	97.96888261
rs217386	7	44600695	A	G	0.4077	-0.0363	0.0038	1.20E-19	91.25210561
rs710887	7	44709372	C	T	0.7124	0.029	0.0042	5.32E-11	47.6753898
rs7791240	7	44602589	C	T	0.09103	0.0425	0.0065	1.84E-10	42.75116799

Asterisk ([#]) represents SNPs selected when the linkage disequilibrium (LD) parameter changes from $r2<0.3$ to $r2<0.1$.
