# Supplementary material for: Causal Association Between Cholesterol-Lowering Drugs and Diabetic Microvascular Complications: A Drug-Target Mendelian Randomization Study
Source: J Diabetes Res. 2025 Feb 28;2025:3661739. doi: 10.1155/jdr/3661739 (PMC11986941; doi:10.1155/jdr/3661739)
Supplement: Supporting Information 3 — Table S3: the effect of HMGCR, PCSK9, and NPC1L1 inhibitors on microvascular complications of diabetes. [file 3661739.f3.pdf]

Table S3. The effect of HMGCR, PCSK9 and NPC1L1 inhibitor on microvascular complications of diabetes.

Outcome	Target	Method	nSNP	Pval	OR	or_lci95	or_uci95
Coronary heart disease	HMGCR	MR Egger	23	0.001190434	0.515291999	0.36424565	0.728974647
		Weighted median	23	2.12E-09	0.67535665	0.593937608	0.767936898
		Inverse variance weighted	23	3.98E-11	0.73400686	0.669647647	0.804551576
		Simple mode	23	0.00146499	0.667550449	0.53681961	0.830117966
		Weighted mode	23	6.85E-05	0.663443057	0.562867732	0.78198956
	PCSK9	MR Egger	12	0.00397397	0.576643603	0.431460195	0.770680237
		Weighted median	12	4.20E-08	0.599631379	0.49942953	0.719936985
		Inverse variance weighted	12	3.54E-12	0.600368024	0.519955849	0.693216096
		Simple mode	12	0.005783017	0.58494639	0.429952479	0.795814181
		Weighted mode	12	7.79E-04	0.601347913	0.483934509	0.747248451
	NPC1L1	MR Egger	4	0.979505165	1.02369259	0.210191126	4.985683929
		Weighted median	4	0.002576666	0.590779981	0.419559562	0.831874704
		Inverse variance weighted	4	3.45E-04	0.583396615	0.434302657	0.783673792
		Simple mode	4	0.11160463	0.593288484	0.375249919	0.938018123
		Weighted mode	4	0.108598416	0.608596585	0.395874291	0.935624798
Diabetic nephropathy	HMGCR	MR Egger	22	0.553509004	1.29837439	0.555384636	3.035330736
		Weighted median	22	1.84E-05	2.005335186	1.458482332	2.757228605
		Inverse variance weighted	22	5.55E-08	1.881580368	1.497893356	2.363549224
		Simple mode	22	0.002709609	2.377096502	1.442616155	3.916903161
		Weighted mode	22	0.001435411	2.08680565	1.408421168	3.091942895
	PCSK9	MR Egger	20	0.812307591	1.035343035	0.780527326	1.373347435
		Weighted median	20	3.47E-01	1.117386445	0.886482143	1.408434991
		Inverse variance weighted	20	9.18E-03	1.300212551	1.067176502	1.584135966
		Simple mode	20	0.93442738	0.978323178	0.584430099	1.637691561
		Weighted mode	20	0.430361758	1.096643258	0.876202662	1.37254369
	NPC1L1	MR Egger	4	0.339930627	9.029891284	0.280737391	290.4455881
		Weighted median	4	0.396060002	0.699899337	0.307007947	1.595590885
		Inverse variance weighted	4	0.574047114	0.828756431	0.430515036	1.59538498
		Simple mode	4	0.416179068	0.520441437	0.133514365	2.028690237
		Weighted mode	4	0.47348344	1.527730681	0.553137979	4.219491556
Diabetic retinopathy	HMGCR	MR Egger	22	0.319574549	1.438375446	0.715689866	2.890810705
		Weighted median	22	2.17E-07	1.937269568	1.50871923	2.487549245
		Inverse variance weighted	22	6.28E-11	1.857814337	1.542931956	2.236958083
		Simple mode	22	0.001670434	2.01192828	1.375510639	2.94280196
		Weighted mode	22	0.000265685	1.955189824	1.447791363	2.640413078
	PCSK9	MR Egger	20	0.951407831	1.006306452	0.824387192	1.228370219
		Weighted median	20	0.641428498	1.04129076	0.878247149	1.234602865
		Inverse variance weighted	20	0.676546367	1.027918441	0.903180231	1.169884243
		Simple mode	20	0.645661408	0.93583674	0.708568423	1.235999764
		Weighted mode	20	0.625507393	1.041895514	0.885942435	1.225301126
	NPC1L1	MR Egger	4	0.489624368	3.181317156	0.213263394	47.45670897
		Weighted median	4	0.034647433	0.519655193	0.283104821	0.953857013
		Inverse variance weighted	4	0.011466344	0.489126802	0.280956869	0.8515365
		Simple mode	4	0.502729695	0.681032211	0.252749329	1.835038985
		Weighted mode	4	0.370783384	0.671302223	0.319062672	1.412408013
Diabetic neuropathy	HMGCR	MR Egger	22	0.570242511	1.480824121	0.390416871	5.616663217
		Weighted median	22	1.53E-08	3.085121664	2.088249866	4.557872042
		Inverse variance weighted	22	1.14E-07	2.625621296	1.837717759	3.751330778
		Simple mode	22	0.0005011	3.269892685	1.858145911	5.754229583
		Weighted mode	22	0.000132257	3.108052449	1.930311795	5.004367713
	HMGCR[#]	MR Egger	12	0.342897911	2.005431175	0.509679875	7.890745532
		Weighted median	12	1.05E-04	2.626193662	1.612093226	4.278222274
		Inverse variance weighted	12	3.14E-06	2.741114243	1.793907737	4.188458046
		Simple mode	12	0.00590142	3.469694186	1.694617238	7.104127985
		Weighted mode	12	0.001991754	2.822733653	1.703448036	4.677468936
	PCSK9[#]	MR Egger	20	0.649242029	1.076264192	0.788244989	1.469523596
		Weighted median	20	0.189834125	1.189905776	0.917538157	1.54312466
		Inverse variance weighted	20	0.000986041	1.40469399	1.1475728	1.719424864
		Simple mode	20	0.240306339	1.412412967	0.808133742	2.468539901
		Weighted mode	20	0.165582187	1.20212818	0.936009504	1.543907573
	PCSK9	MR Egger	16	0.437325338	1.138143592	0.828776751	1.562991281
		Weighted median	16	0.260264284	1.173472136	0.888215176	1.550341505
		Inverse variance weighted	16	0.009168156	1.324244058	1.072073871	1.635729003
		Simple mode	16	0.411772982	1.253917806	0.741546943	2.120310628
		Weighted mode	16	0.262970293	1.174874238	0.895451955	1.541489153
	NPC1L1	MR Egger	4	0.705880883	2.555546234	0.037350303	174.8531092
		Weighted median	4	0.124029814	0.474796169	0.183769876	1.226704872
		Inverse variance weighted	4	0.095418857	0.506961187	0.228136276	1.126561938
		Simple mode	4	0.222230902	0.312969741	0.071045601	1.378692792
		Weighted mode	4	0.828551766	0.872642651	0.281643253	2.703793495

Asterisk ([#]) represents SNPs selected when the linkage disequilibrium (LD) parameter changes from r2<0.3 to r2<0.1.
